# Serum Klotho as a marker for early diagnosis of acute kidney injury after cardiac surgery

**DOI:** 10.2478/jomb-2019-0024

**Published:** 2020-01-23

**Authors:** Aleš Jerin, Osama F. Mosa, Jurij M. Kališnik, Janez Žibert, Milan Skitek

**Affiliations:** 1 University Medical Centre Ljubljana, Institute of Clinical Chemistry and Biochemistry, Slovenia; 2 Umm Al Qura University, Health Science College at Lieth, Department of Public Health, KSA; 3 University Medical Centre Ljubljana, Department of Cardiac Surgery, Slovenia; 4 University of Ljubljana, Faculty of Health Sciences, Slovenia

**Keywords:** acute kidney injury, cardiac surgery, vreatinine, klotho protein, Klotho, kreatinin, operacija srca, akutno oštećenje bubrega

## Abstract

**Background:**

Early diagnosis of acute kidney injury (AKI) after cardiac surgery is based on serum creatinine which is neither a specific nor a sensitive biomarker. In our study, we investigated the role of serum Klotho in early prediction of AKI after cardiac surgery using cardiopulmonary bypass (CPB).

**Methods:**

The included patients were classified into three groups according to AKI stages using KDIGO criteria. The measurements of creatinine and Klotho levels in serum were performed before surgery, at the end of CPB, 2 hours after the end of CPB, 24 hours and 48 hours postoperatively.

**Results:**

Seventy-eight patients were included in the study. A significant increase of creatinine levels (p<0.001) was measured on the first day after the surgery in both AKI groups compared to the non-AKI group. However, a significant difference between AKI-2 and AKI-1 groups (p=0.006) was not measured until the second day after the operation. Using decision trees for classification of patients with a higher or lower risk of AKI we found out that Klotho discriminated between the patients at low risk of developing more severe kidney injury in the first hours after surgery and the patients at high risk better than creatinine. Adding also the early measurements of creatinine in the decision tree model further improved the prediction of AKI.

**Conclusions:**

Serum Klotho may be useful to discriminate between the patients at lower and the patients at higher risk of developing severe kidney injury after cardiac surgery using CPB already in the first hours after surgery.

## Abstract (sr)

**Uvod:**

Rana dijagnoza akutnog oštećenja bubrega (AKI) posle operacije srca se zasniva na kreatininu u serumu koji nije ni specifičan ni osetljiv biomarker. U našoj studiji, istraživali smo ulogu serumskog Klotho-a u ranom predviđanju AKI nakon operacije srca koristeći kardiopulmonarni bajpas (CPB).

**Metode:**

Uključeni pacijenti su klasifikovani u tri grupe u skladu sa AKI fazama po KDIGO kriterijumu. Merenje kreatinina i Klotho-a u serumu je bilo izvedeno pre operacije, krajem CPB, dva sata nakon završetka CPB, 24 sati i 48 sati postoperativno.

**Rezultati:**

U studiju je uključeno sedamdeset osam pacijenata. Povišenje nivoa kreatinina prvi dan posle operacije je bilo značajno (p < 0,001) u obe AKI grupe u poređenju sa non-AKI grupom, a značajna razlika između grupa AKI-2 i AKI-1 (p = 0,006) bila je izmerena tek drugi dan posle operacije. Upotreba stabla odlučivanja za klasifikaciju pacijenata sa manjim i sa većim rizikom od razvijanja oštećenja bubrega pokazala je da je Klotho u prvim satima nakon operacije bolji od kreatinina u diferencijaciji između pacijenata sa manjim i pacijenata sa većim rizikom od oštećenja bubrega. Predviđanje razvoja težeg oštećenja bubrega bilo je još bolje sa dodatnim uključivanjem ranih rezultata kreatinina u model.

**Zaključak:**

Serumski Klotho može biti koristan za diferencijaciju između pacijenata sa manjim i pacijenata sa većim rizikom od razvijanja težeg oštećenja bubrega posle operacije srca koristeći CPB već u prvim satima nakon operacije.

## Introduction

Acute kidney injury (AKI) that can occur after the surgery is characterized by rapid progressive complications and adverse outcomes, which are raising morbidity and mortality rates high above ordinary rates observed in major surgeries [1] [2] [3]. Criteria for classification of AKI stages have evolved over the years, and recently the Kidney Disease Improving Global Outcomes (KDIGO) consensus conference on AKI recommended re-arrangement and harmonization of classification criteria for AKI by using new KDIGO criteria [4]. Although classifications and definitions of AKI are well established, they are insufficient for early diagnosis of AKI as they mainly rely on serum creatinine which is neither a specific nor a sensitive marker; its baseline is affected by sex, age, dietary status, muscle mass, medications and it is raised eventually when 50% of kidney function is already lost [5]. Early prediction and prognosis of AKI are inevitable to improve the treatment. Therefore, novel markers are needed, which would be used side by side with common traditional biomarkers like creatinine. The role of cystatin C, neutrophil gelatinaseassociated lipocalin, kidney injury molecule 1, interleukin-18 and other novel early markers of AKI has been evaluated in several studies [6] [7] but for some potential early markers like Klotho, the data are still very limited.

Klotho is originally produced as a single pass transmembrane protein with a small extracellular domain. After the removal of the extracellular domain by secretases, two types of Klotho with different functions are released [8] [9]. While membrane-bound Klotho acts as a receptor for fibroblast growth factor-23, secreted soluble Klotho regulates several ion channels [10] [11], growth factors like IGF-1 and is also involved in oxidative stress [12]. Klotho is expressed in high concentration in renal tubules, but it can also be found in the brain and to a lower extent also in the parathyroid gland and the heart [13] [14] [15].

The pathophysiological mechanism by which Klotho is involved in kidney injury is still puzzling. The potential role of Klotho in AKI was proposed by Hu et al. [16]. The model assumed that exposure to acute ischemia, oxidative stress and some other factors is a potential reason for the decrease of Klotho levels. Lower Klotho levels may be an aggravating factor of renal damage. If renal damage is mild, the kidney tissue will recover, while in the case of more severe injuries, regeneration could stop and subsequent fibrosis could than even worsen renal Klotho insufficiency. Several protective actions of Klotho were proposed including reduction of oxidative stress through regulation of expression of mitochondrial superoxide dismutase and catalase [17] and prevention of apoptosis through activation of heat shock protein-70 [18].

Recent studies of AKI on rodent models revealed an initial reduction of renal, blood and urinary Klotho levels in response to renal tubular injury with a possibility of reversing after the regeneration of kidney function [19] [20], indicating an association of AKI with a state of endogenous Klotho deficiency. In human studies, the decrease of renal Klotho expression in AKI was found to be associated with the severity of the kidney injury [21].

In our study, we aimed to assess the significance of serum Klotho for early diagnosis of AKI after cardiac surgery using cardiopulmonary bypass (CPB).

## Materials and Methods

### Subjects

The present pilot study was conducted on patients who were admitted to Department of Cardiovascular Surgery at the University Medical Center Ljubljana for elective cardiac surgery with CPB and was a part of a larger study of kidney function. Preoperative kidney function was normal in all studied patients. The exclusion criteria were a history of kidney disease including diabetic nephropathy, renal transplantation, malignancy, autoimmune diseases and pregnancy. Based on the experience from the previous work [22], the included patients were classified into three groups according to AKI stages instead of simple classification into AKI and non-AKI groups. The classification was done based on KDIGO criteria [4] using the concentration of creatinine in serum at different time-points. The study was carried out according to the declaration of Helsinki. The National Medical Ethics Committee of the Republic of Slovenia approved the study protocol, and informed consent was obtained from all study participants before data collection.

### Methods

Blood samples were collected before surgery, at the end of CPB, 2h after the end of CPB, 24h and 48h postoperatively. Samples were collected in tubes without additives; following centrifugation aliquots of serum were frozen and stored at -20 °C until analysis. The measurement of Klotho levels in serum was performed by ELISA kit (Elabscience Ltd, Wuhan, China) with detection limit 0.19 μg/L and CV < 10% while the concentration of serum creatinine was measured using automatic kinetic Jaffe reaction (Siemens Healthcare Diagnostics Inc., Newark, DE, USA), which is traceable to IDMS reference method. Estimated glomerular filtration rates for all patients were calculated using the MDRD equation [23].

### Statistical analysis

Data were expressed as mean ± standard deviation. The time-series collection of blood samples were analyzed by using the repeated measures ANOVA test with the post-hoc analysis made by Bonferroni's correction procedure. Significance was set at p < 0.05. Decision trees including early measurements of Klotho, creatinine or the combination of both parameters as independent variables were used for early classification of patients into two groups: patients at higher risk of AKI and patients at a lower risk. Areas under the ROC curve were calculated for these classifications. Statistical analyses were performed with the SPSS software (IBM SPSS Statistics, version 20).

## Results

**Figure 1:**
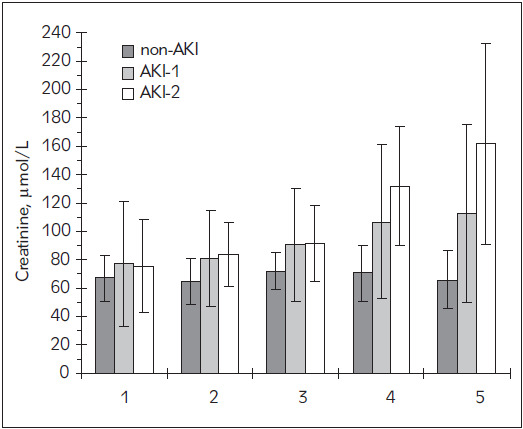
The estimated marginal means of serum creatinine Legend: 1: preoperative; 2: at the end of CPB; 3: 2 hours after the end of CPB; 4: 24 hours postoperatively; 5: 48 hours postoperatively. Error bars correspond to standard errors of estimated marginal means in the repeated measures ANOVA.

**Figure 2:**
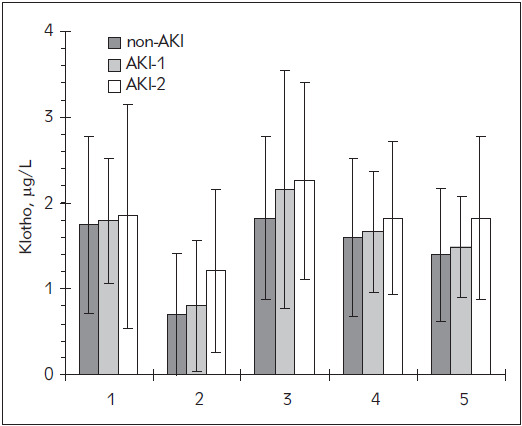
The estimated marginal means of serum Klotho concentrations Legend: 1: preoperative; 2: at the end of CPB; 3: 2 hours after the end of CPB; 4: 24 hours postoperatively; 5: 48 hours postoperatively. Error bars correspond to standard errors of estimated marginal means in the repeated measures ANOVA

Seventy-eight patients were included in the study; they were classified into three groups according to AKI stages. The classification was based on changes in serum creatinine using KDIGO criteria as shown in Table 1. Creatinine levels are shown in Figure 1. The preoperative concentrations of serum Klotho were not significantly different between the groups. During the surgery Klotho levels decreased in all groups, reaching the minimum of approximately half of the preoperative values at the end of CPB. Two hours after CPB, its levels increased above the preoperative baseline and ultimately decreased back within 48 hours as shown in Figure 2 and Table 2. Although at the end of CPB there was a great difference in Klotho levels between the groups (p = 0.086), the differences were not significant at any time-point. The levels were not significantly different between the groups before surgery. A great increase (p < 0.001) was measured 24 hours after the surgery in AKI-2 and AKI-1 groups compared to the non-AKI group, but the increase in AKI-2 group became significantly higher than in AKI-1 group (p = 0.006) no earlier than 48 hours after surgery.

**Table 1:** A summary of clinical characteristics of patients in all three groups: non-AKI, AKI-1 and AKI-2

	Non-AKI	AKI-1	AKI-2
Number of patients and Gender (m/f)	26 (13/13)	30 (15/15)	22 (12/10)
Age (yrs)	68.3 ± 11.1	71.8 ± 12.7	74.0 ± 7.7
Preoperative eGFR (mL/min/1.73 m^2^)	102.7 ± 26.2	119.9 ± 59.9	113.8 ± 47.9
Preoperative Creatinine (μmol/L)	67.0 ± 15.7	77.2 ± 44.1	75.3 ± 33.0
Diabetes mellitus (none/oral/insulin)	17/6/3	21/5/4	15/5/2
Arterial hypertension (y/n)	6/20	5/25	5/17
Cardiopulmonary bypass time (min)	93.6 ± 32.4	95.5 ± 36.8	106.7 ± 36.7
Aortic cross-clamping (min)	72.3 ± 30.4	71.2 ± 30.9	81.5 ± 29.9
RBC transfusion (units)	1.8 ± 2.3	2.6 ± 3.6	2.2 ± 1.8
Fresh frozen plasma (mL/kg)	1.3 ± 1.8	2.2 ± 3.3	1.0 ± 1.4
Intensive care unit stay (days)	5.0 ± 5.0	8.9 ± 9.6	6.5 ± 4.6
Hospital stay (days)	11.3 ± 7.3	17.7±23.6	11.8±7.0

**Table 2:** The estimated marginal means and standard errors of serum creatinine and Klotho concentrations at different timepoints

	Non-AKI	AKI-1	AKI-2
Serum creatinine (μmol/L)			
Preoperative	67.0 ± 15.7	77.2 ± 44.1	75.3 ± 33.0
At the end of CPB	64.6 ± 16.3	80.6 ± 33.5	83.3 ± 22.7
2 hours after the end of CPB	72.0 ± 13.0	90.7 ± 39.6	91.5 ± 26.5
24 hours postoperatively	70.6 ± 19.5	106.6 ± 54.1	131.7 ± 41.7
48 hours postoperatively	65.8 ± 20.6	112.8 ± 63.0	162.1 ± 70.7
Serum Klotho (μg/L)			
Preoperative	1.75 ± 1.03	1.80 ± 0.73	1.85 ± 1.30
At the end of CPB	0.70 ± 0.72	0.80 ± 0.76	1.21 ± 0.95
2 hours after the end of CPB	1.83 ± 0.95	2.16 ± 1.38	2.26 ± 1.15
24 hours postoperatively	1.60 ± 0.92	1.67 ± 0.71	1.83 ± 0.89
48 hours postoperatively	1.40 ± 0.78	1.49 ± 0.60	1.83 ± 0.95

**Figure 3:**
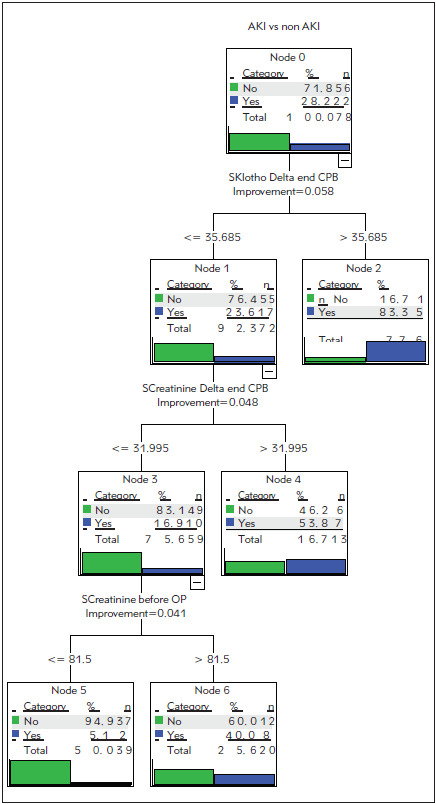
Classification of patients using decision tree, which includes the concentrations of Klotho and creatinine in serum Group 0: non-AKI + AKI-1; group 1: AKI-2

To discriminate between patients at low risk of developing severe kidney injury after surgery and the patients at high risk, decision trees were used. Patients were classified into the group at higher risk of AKI (AKI-2) and the group at lower risk (non-AKI + AKI-1). Decision trees were made using only Klotho or only creatinine concentrations and also using the combination of both markers. Figure 3 shows the decision tree, which was built using Klotho and creatinine concentrations at the end of the surgery, two hours after the end of surgery and also the differences from the preoperative concentration. At the beginning, in the Node 0, we started with a group of 22 patients classified as AKI-2 according to KDIGO criteria. The optimal division at the first level was achieved by selecting the parameter delta Klotho concentration at the end of CPB at value 35.685. In the first and second subgroup 23.6 % and 16.7 % of patients were classified as AKI-2, respectively. The second division was made in the first group, where the optimal threshold was estimated based on the parameter delta Creatinine concentration at the end of CPB at value 31.995. The last division in this decision tree was made with the optimally chosen parameter of Creatinine concentration before the operation.

**Figure 4:**
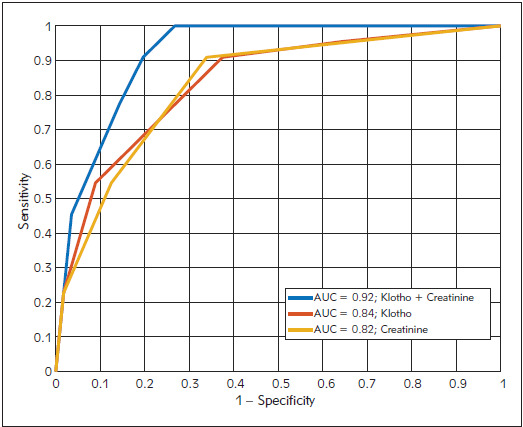
Areas under the ROC curve for Klotho, creatinine and the combination of both parameters

The summary results for all three decision trees are presented in Table 3. The input parameters in the first decision tree were Klotho concentrations at the end of the surgery, two hours after the end of surgery and the differences from the preoperative concentration. The outputs of the classification were probabilities of the patient to belong to AKI-2 or non-AKI+AKI-1 group. In this case, we achieved the overall accuracy of 80.8 %. The input parameters in the second decision tree were creatinine concentrations at the end of the surgery, two hours after the end of surgery and also the differences from the pre-operative concentration. The classification results were produced in the same way as in the Klotho case. The overall accuracy of 74.4 % was achieved. The best overall accuracy (83.3 %) was achieved in the case of the decision tree where the input parameters were both Klotho and creatinine concentrations at the end of the surgery, two hours after the end of surgery and also the differences from the preoperative concentrations. Areas under the ROC curve for Klotho, creatinine and the combination of both parameters were 0.84, 0.82 and 0.92, respectively (Figure 4).

**Table 3:** Classification of patients using decision trees including Klotho and creatinine concentrations at the end of the surgery, two hours after the end and also the differences from the preoperative concentration

Observed	Predicted		
	Non-AKI + AKI-1	AKI-2	Percent correct
Klotho			
Non-AKI + AKI-1	51	5	91.1 %
AKI-2	10	12	54.5 %
Overall percentage	78.2 %	21.8 %	80.8 %
Creatinine			
Non-AKI + AKI-1	40	16	71.4 %
AKI-2	4	18	81.8 %
Overall percentage	56.4 %	43.6 %	74.4 %
Klotho + creatinine			
Non-AKI + AKI-1	45	11	80.4 %
AKI-2	2	20	90.9 %
Overall percentage	60.3 %	39.7 %	83.3 %

## Discussion

AKI following cardiac surgery can be reversible, but in worse cases, it can progress into irreversible stages. In our study, we evaluated serum levels of Klotho as a possible early marker of AKI after cardiac surgery using CBP. Immediately after the surgery, Klotho levels were drastically decreased in all groups of patients indicating the influence of the surgical procedure on the kidney. To evaluate the significance of serum Klotho as a marker of AKI in the first hours after surgery, we used decision trees for classification of patients with a higher and lower risk of AKI. Klotho discriminated between the patients at low risk of developing severe kidney injury after the surgery and the patients at high risk better than creatinine. Combination of both parameters in the decision tree model further improved the early prediction of AKI.

Serum creatinine exhibited a significant difference between groups on the first day after the operation, but the levels were not significantly higher in AKI-2 group than in AKI-1 group until the second day after the operation. Since the classification of patients to the groups was based on creatinine changes, the observed differences between the groups on the second day after surgery were expected.

The decrease of Klotho after the operation could be partially caused by the effect of CPB used during the surgery, which can lead to volume depletion, decreased renal blood flow and renal ischemic injury [24] [25]. Liu YJ et al. [26] found out that Klotho levels in serum samples of patients who developed AKI after cardiac valve replacement were significantly reduced immediately after the surgery and restituted four hours to three days postoperatively while no decrease was measured in samples of patients who did not develop AKI. Studies on animal models also found a remarkable decrease in plasma and urinary Klotho within three hours after the renal insult [27]. Our measurements two hours after the end of CPB showed that Klotho levels increased above the preoperative levels in all groups. These initial changes were followed by a continuous decrease of serum Klotho levels on the first and the second postoperative day, indicating only a transient stimulus for increased Klotho production. Considering the protective role of Klotho, which was described by several researchers in AKI [20] [27] as well as in chronic kidney disease [28] [29], the fast increase after the initial stimulus might be explained as a saving response to prevent further inflammation, apoptosis and attenuation of renal injury. This is adding some new perspective to the possible use of Klotho: it might prove to be not only an early marker of AKI but also a therapeutic agent [19].

To our knowledge, there is only one study describing the role of serum Klotho as a marker of AKI after cardiac surgery. The researchers found a significant difference in serum Klotho values between the non-AKI group and AKI group in the first hours after surgery [26], but they did not divide AKI patients to subgroups according to AKI stages. Therefore, it is not possible to directly compare the two studies. Nevertheless, the conclusion that serum Klotho may be useful for early discrimination between the patients at low risk of developing severe kidney injury after surgery and the patients at high risk (AKI stage 2) is supported by the results of both studies.

When investigating the role of Klotho in kidney injury, some researchers focused on Klotho levels in human urine and renal tissue. The decrease of Klotho values in renal tissue and urine samples after acute kidney injury was confirmed in animal studies [19] and association of urinary Klotho with nephron function in chronic kidney disease was also reported in human studies [29]. The role of urinary Klotho as a marker of AKI after the surgical procedure has not yet been confirmed in human studies, and some authors suggested that urinary Klotho is not a good candidate for AKI marker [30]. In contrast to urinary Klotho, histological examinations revealed the correlation of renal Klotho expression and the severity of AKI regardless of the etiology [21] suggesting the potential of Klotho as a marker of AKI severity.

Although the results of this pilot study show the potential role of Klotho in early prediction of AKI after cardiac surgery using CPB, we should be aware of several limitations, especially when using the decision trees. In this study, the same population of patients was used to set the model and to calculate the results. Future studies are needed to independently validate the proposed statistical approach. It is also worth mentioning that at the present moment, Klotho is still under research and therefore the reference ranges of serum and urine levels are not clearly defined. As the methods for the measurement of Klotho levels are not easily comparable, the use of any cut-offs for decision making would be limited.

## Conclusion

Using the decision trees models, serum Klotho may be useful to identify patients at low risk of developing more severe kidney injury after cardiac surgery using CPB and separate them from patients at high risk already in the first hours after surgery.

Adding also the results of early measurements of creatinine to decision trees models could further improve the prediction of AKI.

### Conflict of interest statement

The authors stated that they have no conflicts of interest regarding the publication of this article.
